# *HOXB13*, a high-risk prostate cancer gene, also confers risk for breast cancer: novel variants of clinical significance, especially in hormone-positive patients

**DOI:** 10.1038/s10038-026-01481-y

**Published:** 2026-05-22

**Authors:** Filiz Ozen, Zeynep Yegin, Diyar Sayit

**Affiliations:** 1 Medical Genetics Clinic, Istanbul Göztepe Prof. Dr. Süleyman Yalçın City Hospital, Istanbul, Turkey; 2 Medical Laboratory Techniques Program, Vocational School of Health Services, Sinop University, Sinop, Turkey

**Keywords:** Predictive markers, Cancer genetics

## Abstract

Germline mutations in high-risk genes, such as *BRCA1* and *BRCA2*, are primarily responsible for inherited breast cancers, while mutations in moderate-risk genes also increase susceptibility. *HOXB13* is established as a high-risk gene for prostate cancer (PCa), but few studies have explored its role in breast cancer. Here, we report, for the first time, novel *HOXB13* variants identified in breast cancer patients, suggesting a potential association that warrants further investigation. This retrospective cohort study was conducted at the Medical Genetics Laboratory of Göztepe Prof. Dr. Süleyman Yalçın City Hospital, Turkey, between 2022 and 2025. A total of 4198 individuals who underwent hereditary cancer panel testing were screened. Targeted next-generation sequencing was performed using a Custom Hereditary Cancer (cHCS) panel on the Illumina NextSeq® platform. Raw sequencing data were analyzed using Dragen v3.6 and Sophia DDM pipelines. Among 4198 individuals, 1527 had a confirmed breast cancer diagnosis. Eleven patients harbored variants of uncertain significance (VUS) in *HOXB13*. Hormone-negative patients (*n* = 3) carried c.269del, c.766 T > C, and c.309_311del variants. Among hormone-positive patients (*n* = 8), two carried c.309_311del, one had c.404 G > A, and five carried c.728 A > G (p.K243R), the most frequent variant in this subgroup. The *HOXB13* variant spectrum in the Turkish population differed from previously reported data. Novel *HOXB13* variants, particularly c.728 A > G (p.K243R), may represent candidate variants of interest that require further validation before any clinical application. These findings highlight a potential association between *HOXB13* variants and breast cancer, particularly in hormone receptor-positive patients, and emphasize the need for larger and functionally validated studies.

## Introduction

Breast cancer is a complex disease with varying degrees of risk among women and genetic liability plays a significant role in the etiology of the disease [1]. It is both the most commonly diagnosed and the second leading cause of cancer death among Western women. Risk ratios for breast cancer varies from 1.80 for one affected relative to 3.90 for three or more affected relatives underpinning the potential effect of family history [2]. Several germline variants which explain approximately half of the total genetic heritability have been reported [3]. *BRCA1* and *BRCA2* which are the major breast cancer susceptibility genes were identified in the 1990s and they are responsible for 15–20% of the inherited breast cancers. Germline mutations in these two high-risk genes constitute cumulative lifetime breast cancer risks by age 70 of 65 and 45%, respectively [1, 2]. Besides, some other mutations in moderate-risk genes such as *ATM*, *CHEK2*, *PALB2*, and *RAD50* constitute 2- to 4-fold increased breast cancer risks. Many common low-risk alleles may also display small but additive effects which exhibit important effects in terms of individualized treatment regimens [2].

The homeobox transcription factor (*HOX*) gene family belongs to a larger homeobox superfamily of transcription factors characterized by a highly-conserved DNA-binding domain. *HOX* genes which are 39 in total are subdivided into four clusters (A, B, C, D) on chromosomal locations 7p15, 17q21, 12q13, and 2q31, respectively [4]. *HOXB13* is composed of two exons and one intron; exon 1 includes two MEIS (myeloid ecotropic viral integration site) binding domains while exon 2 contains a homeodomain and both domains are highly conserved among vertebrates [5]. *HOX* genes play a significant role in animal embryonic development and may promote proliferation of stem and progenitor cells [4]. *HOXB13* has a broad expression spectrum from low levels in colon and rectum to high levels in the normal prostate gland and prostate cancer. Indeed, after screening study for more than 200 genes in the 17q21-22 region from 94 unrelated patients with prostate cancer from families selected for linkage to the candidate region in 2012, *HOXB13* was introduced to the literature as a high-risk gene for prostate cancer (PCa) liability [6]. Especially, a rare but recurrent mutation, G84E (rs138213197), has been frequently observed in germline DNA of European descent men with prostate cancer [7–10]. HOXB13 has been shown to regulate transcriptional activity of the androgen receptor (AR) critical to prostate tumor growth. Moreover, HOXB13 can promote prostate cancer by up-regulating NF-kappaB pathway which induces tumor invasion and metastasis [9, 11]. A significant association between germline G84E mutation and colorectal cancer was also reported [12].

*HOXB13* has also drawn attention in breast cancer susceptibility and management studies since it was reported that HOXB13 alone or HOXB13: IL17BR ratio could predict the benefit of adjuvant endocrin therapy with tamoxifen in primary breast cancer [13]. Besides, the above mentioned high-risk mutation for prostate cancer was also found to be associated with risk of *BRCA*1/2 wild-type familial breast cancer [14]. Despite these potential roles of HOXB13, the studies aiming to explore *HOXB13* germline mutations in breast cancer are quite limited and in need of exploring and standardizing new mutations in different populations to better analyze sub-cohorts and individualize treatment regimens. In our study approach, we performed clinical exome sequencing (CES) to identify *HOXB13* variants in breast cancer patients, with a particular focus on hormone receptor-positive cases.

## Materials & methods

### Patients and ethical considerations

**Fig. 1:**
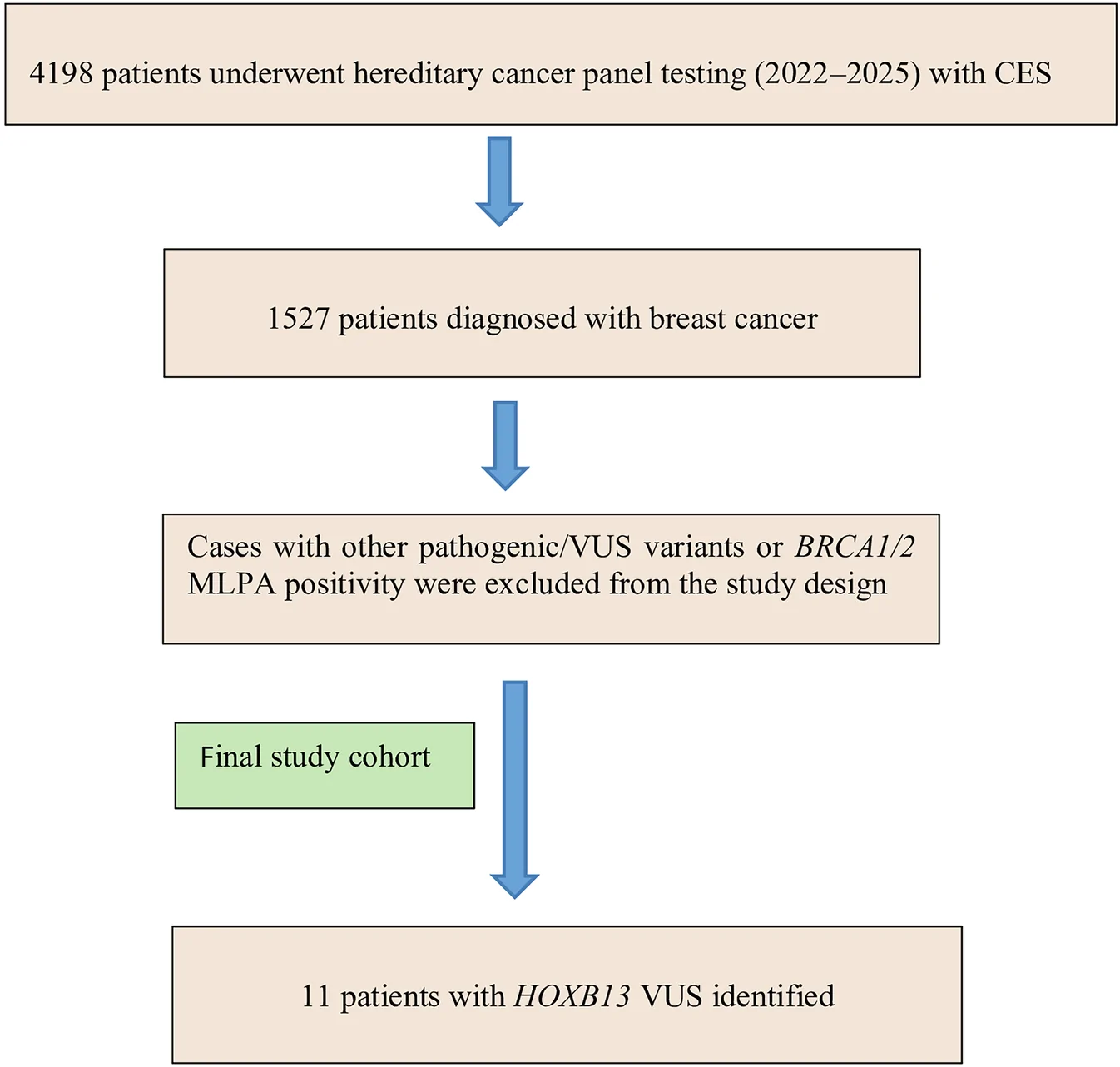
Flowchart of the genetic algorithm applied in this study

The study was approved by the Institutional Medical Ethics Committee of Istanbul Göztepe Prof. Dr. Süleyman Yalçın City Hospital (2025/0298). Informed consent was obtained from the patients. The study procedures have been performed in accordance with the ethical standards as laid down in the 1964 Declaration of Helsinki and its later amendments or comparable ethical standards. This retrospective, single-center study was conducted at the Medical Genetics Laboratory of Göztepe Prof. Dr. Süleyman Yalçın City Hospital between January 2022 and January 2025. During this period, a total of 4198 individuals who underwent hereditary cancer panel testing were retrospectively screened. Among these individuals, 1527 patients had a confirmed diagnosis of breast cancer. Patients harboring variants of uncertain significance (VUS) in the *HOXB13* gene were identified within this subgroup. Eleven breast cancer patients carrying *HOXB13* VUS variants were identified. A flowchart for the design and steps of the study is shown in Fig. 1.

### Inclusion and exclusion criteria

Inclusion criteria comprised: (i) availability of hereditary cancer panel sequencing data; (ii) detection of a VUS in the *HOXB13* gene; (iii) confirmed diagnosis of breast cancer and (iv) absence of other pathogenic, likely pathogenic, or clinically explanatory VUS variants. Patients were excluded if variants in genes other than *HOXB13* were identified that could explain the clinical phenotype. All breast cancer patients underwent *BRCA1* and *BRCA2* copy number analysis using multiplex ligation-dependent probe amplification (MLPA), and only MLPA-negative cases were included in the final analysis.

### Clinical and pathological data collection

Demographic and clinical information was obtained retrospectively from the hospital electronic medical record system. Tumor hormone receptor status, including estrogen receptor (ER), progesterone receptor (PR), and HER2 expression was extracted from pathology reports. Tumors were classified as hormone receptor–positive or –negative based on standard pathological criteria. All relevant clinical, pathological, and molecular findings were summarized in Table 1.

**Table 1:** Clinical, pathological, and immunohistochemical characteristics of individuals carrying *HOXB13* variants

Case	Age at diagnosis (y)	Primary diagnosis	Breast tumor pathology subtype	ER	PR	HER2	Ki-67	*HOXB13* variant	Variant class	Notes / other neoplasms	Family history
**1**	45	Breast cancer	Not available	Neg	Neg	Pos	NA	c.269del	Frameshift del	HR– / HER2+ breast cancer	Maternal cousin, BC (28)
**2**	42	Breast cancer	High-grade DCIS	Neg	Neg	NA	NA	c.766 T > C	Missense	Right breast; excisional biopsy	Father, prostate ca (72)
**3**	39	Breast cancer	Mixed invasive (≈75% cribriform; ≈25% IDC-NOS) + DCIS ( ~ 20%)	Pos	Pos	Neg (ISH − )	37%	c.309_311del	In-frame del	Tumor 2.5 cm; pT2 pN0(sn)	No FH
**4**	54	Breast cancer	Not available (tru-cut; limited)	Neg	Neg	Eq/heterog; SISH inconcl.	90%	c.309_311del	In-frame del	Repeat HER2 testing on surgical specimen advised	Distant maternal relative, BC
**5**	70	Breast cancer	Low-grade DCIS; intraductal papilloma with ADH/low-grade DCIS foci	Pos (A8)	Pos (A7)	NA	NA	c.309_311del	In-frame del	Re-excision; margins negative	Niece, BC (35, deceased)
**6**	45	Breast cancer	Not available	Pos (A8)	Pos (A8)	NA	NA	c.728 A > G	Missense	HR+ breast cancer	No FH
**7**	52	Breast cancer	Invasive carcinoma, NOS	Pos (A6)	Pos (A8)	IHC 2 + ; FISH− (1.2)	NA	c.728 A > G	Missense	Stage as reported: pT1c pN2a(sn)	Maternal aunt + cousin, BC
**8**	65	Breast cancer	Not available	Pos (A8)	Neg (A0)	Pos (IHC 3 + )	50–60%	c.728 A > G	Missense	GATA3 + ; high proliferation	Sister, endometrial ca
**9**	52	Breast cancer	Not available	Pos (A8)	Pos (A7)	NA	NA	c.728 A > G	Missense	HR+ breast cancer	Father, prostate ca (60 s)
**10**	55	Breast cancer	Invasive carcinoma, NOS (tru-cut); in situ component present	Pos (A8)	Neg (A0)	Pos (IHC 3 + )	25%	c.728 A > G	Missense	Right breast (4 o’clock); E-cadherin diffuse	Paternal cousin, BC (58)
**11**	36	Breast cancer	Invasive ductal carcinoma (IDC), high-grade	Pos	Neg	Pos (IHC 3 + )	40%	c.404 G > A	Missense	HER2+ breast cancer; high-grade	Mother, BC (40)

*ADH* atypical ductal hyperplasia, *A* allred score, *BC* breast cancer, *DCIS* ductal carcinoma in situ, *ECE* extranodal extension, *Eq* equivocal, *ER* estrogen receptor, *FH* family history, *FISH* fluorescence in situ hybridization, *HR* hormone receptor, *IDC-NOS* invasive ductal carcinoma—no special type, *IHC* immunohistochemistry, *ISH* in situ hybridization, *LG* low grade, *NA* not available, *NG* nuclear grade, *PR* progesterone receptor, *SN* sentinel node, *SISH* silver in situ hybridization

### Genomic DNA extraction and quality control

Peripheral blood samples were collected in EDTA-containing tubes as part of routine clinical testing. Genomic DNA was extracted from peripheral blood leukocytes using standardized silica membrane–based extraction protocols according to the manufacturer’s instructions. DNA concentration and purity were assessed using spectrophotometric measurements. Samples with acceptable purity ratios (A260/280 and A260/230) and sufficient DNA yield were selected for downstream library preparation.

### Target enrichment and library preparation

Targeted next-generation sequencing (NGS) was performed using a Custom Hereditary Cancer (cHCS) panel designed to capture the coding exons and flanking intronic regions of genes associated with hereditary cancer predisposition, including *HOXB13*. Genomic DNA underwent multiplex PCR-based target enrichment to selectively amplify disease-associated regions. Following amplification, sequencing libraries were prepared by ligating platform-specific adapters and unique index sequences. Libraries were purified to remove primer dimers and nonspecific products, quantified, and normalized prior to sequencing.

### Next-generation sequencing

Sequencing was performed on the Illumina NextSeq® platform using sequencing-by-synthesis technology. Indexed libraries were pooled and sequenced using paired-end reads to improve alignment accuracy and variant detection sensitivity. Sequencing quality metrics were monitored throughout the run.

### Sequencing quality metrics and thresholds

A minimum mean coverage depth of ≥100× across target regions was required for sample inclusion. Regions with coverage below 20× were considered insufficient for reliable variant calling. Variant allele frequency (VAF) thresholds were set at ≥20% for heterozygous germline variants and ≥90% for homozygous variants.

### Bioinformatic analysis

Raw sequencing data were processed using Dragen v3.6 and Sophia DDM pipelines. Reads were aligned to the human reference genome hg19. Variant calling for single nucleotide variants (SNVs) and small insertions/deletions (INDELs) was performed, and results were reported in variant call format (VCF). Coverage metrics were calculated for each target region.

### Variant annotation and classification

Variant annotation was performed using Ensembl Variant Effect Predictor (VEP). Transcript prioritization was conducted using the VEP pick-order algorithm. Variants were classified according to American College of Medical Genetics and Genomics (ACMG) guidelines [15], using population frequency data from gnomAD and 1000 Genomes, functional prediction tools, and domain-based analyses.

## Results

We conducted a retrospective cohort study consisting of a total of 4198 individuals who underwent hereditary cancer panel testing at the Medical Genetics Laboratory of Göztepe Prof. Dr. Süleyman Yalçın City Hospital between January 2022 and January 2025. The initial screening panel resulted in 1527 patients with a confirmed diagnosis of breast cancer. Patients with other pathogenic/VUS variants or *BRCA1/2* MLPA positivity were excluded from the study. Patients harboring variants of uncertain significance (VUS) in the *HOXB13* gene were selected and thus the final cohort resulted in 11 patients. Flowchart of the genetic algorithm applied in this study and clinical, pathological, and immunohistochemical characteristics of individuals carrying *HOXB13* variants are shown in Fig. 1 and Table 1, respectively.

The mean age of the breast cancer patients was 50.45 ± 10.54 years (range: 36–70). Three patients were hormone-negative, while the majority (*n* = 8) were hormone-positive. NGS identified *HOXB13* variants in this cohort, some of which have not been previously reported in breast cancer to our knowledge. Variant distribution differed between hormone-negative and hormone-positive patients. Hormone-negative patients (*n* = 3) carried the c.269del, c.766 T > C, and c.309_311del variants. Among hormone-positive patients (*n* = 8), two carried c.309_311del, one had the c.404 G > A missense variant, and the remaining five carried the c.728 A > G missense variant. The details of the clinical, pathological and immunohistochemical characteristics of the individuals and the newly identified variants’ features are presented in Table 1.

To determine whether the *HOXB13* variants identified in our cohort represent population polymorphisms, we interrogated public databases including gnomAD and ClinVar. The c.269del variant was not identified in either database, suggesting that it is absent from currently available population and clinical variant resources. The c.309_311del (p.Ala104del) variant was present in ClinVar and observed at low frequency in gnomAD (AF = 1.86 × 10⁻⁵), indicating that it is rare but not ultra-rare. In contrast, c.404 G > A (p.Gly135Glu), c.728 A > G (p.Lys243Arg), and c.766 T > C (p.Ser256Pro) were all observed at extremely low allele frequencies in gnomAD (6.20 × 10⁻⁷, 4.96 × 10⁻⁶, and 1.24 × 10⁻⁶, respectively), supporting their classification as ultra-rare variants rather than common polymorphisms. Notably, c.728 A > G recurred in multiple individuals within our cohort despite its rarity. Published data indicate marked ethnic heterogeneity in *HOXB13* variation, with the p.Gly84Glu (G84E) variant enriched in European populations, whereas other variants have been described in non-European groups. However, no clear population-specific enrichment was observed for the variants identified in our cohort in gnomAD, suggesting that they do not correspond to known population-specific polymorphisms.

In addition, among the 2671 individuals without a diagnosis of breast cancer in the overall cohort, *HOXB13* variants were identified in 3 cases (0.11%). Two individuals carried the known pathogenic variant c.251 G > A (p.Gly84Glu), one diagnosed with prostate cancer at the age of 65 and the other with ovarian cancer at the age of 54. The third individual, a 34-year-old patient with insulinoma, harbored the c.766 T > C variant, which was classified as a variant of uncertain significance (VUS). No additional recurrent *HOXB13* variants were identified in the non-breast cancer group.

## Discussion

Literature data show that *HOX* genes may act as a both oncogene and tumor suppressor. In 2012, a rare mutation rs138213197 (c.251 G > A; G84E: substition of adenine for guanine in the second position of codon 84, resulting in the replacement of glutamic acid for glycine) in *HOXB13* gene was reported to be associated with increased risk for prostate cancer [6]. Since then, there have been several studies confirming this association with a special emphasis on early-onset and familial or hereditary prostate cancer [9, 11]. Evaluation of G84E in a very large population (5083 patients and 1401 controls) of European descent showed a > 20-fold increased PCa risk among carriers [8]. The frequency of the haplotype is significantly higher in Nordic countries suggesting a founder allele effect in this part of the world, most likely in Finland or Sweden [11, 16]. G84E carriers were found to be more likely to have a positive family history of prostate cancer in a first degree relative when compared with non-carriers [4]. *HOXB13* G84E was found to be associated with a 3.5-fold increased risk of prostate cancer in Swedish population [8]. A similar result was obtained with the largest population study conducted in British men (8652 men diagnosed with PCa and 5252 healthy men) for G84E germline mutation; a 2.93-fold increased risk for PCa was reported and the risk was associated with both family history and young-onset disease [9]. In a Norwegian population study, *HOXB13* G84E and at least 13 risk variants were found to be associated with PCa risk [16]. It must be taken into account that the frequency of the investigated mutations may vary between geographic regions and this issue must be considered for conducting studies in different populations [9]. G84E mutation was not detected in Chinese men though a novel rare and possibly founder mutation in *HOXB13* gene (G135E) was reported to be associated with increased PCa risk in Chinese men [5]. In two population studies conducted in Japanese patients, two deleterious *HOXB13* variants (c.380 T > G/F127C and c.395 G > A/G132E) were identified and European spesific G84E variant was not found. Besides, G132E pathogenic variant was also reported to be associated with younger diagnosis age and family histories of breast, pancreatic, lung, and liver cancers in the largest case-control sequencing study [17, 18]. G132E variant was also detected in Korean men with PCa [19]. It is apparent that the main pathogenic variants of Asian populations seem different from those of European population main variant G84E. This was also the case observed in men of African descent with PCa; a novel *HOXB13* variant rs77179853 (c.853delT, X285K) was associated with increased risk. It was also related with early age at diagnosis and more aggressive cancer risk [20, 21].

*HOXB13* gene has also drawn attention in breast cancer studies. In the adjuvant therapy of estrogen receptor (ER)-positive breast cancer, hormonal therapy with tamoxifen reduces the risk of breast cancer recurrence and decreases mortality. A high HOXB13/interleukin −17B receptor (IL-17 BR) ratio was reported to be associated with increased relapse and death in patients with lymph node-negative, ER-positive patients treated with tamoxifen. These finding reflects the need of alternative therapy tries such as aromatase inhibitors or chemotherapy for women who are at high risk because of HOXB13/IL-17BR ratio [22]. A subsequent study on the other hand reported that HOXB13:IL17BR ratio would also be predictive for lymph node-positive patients (72% of the patients in the study group were lymph node-positive). Overexpression of HOXB13 would result in contributing to tamoxifen resistance and thus a high HOXB13:IL17BR ratio was associated with aggressive tumor features [13]. Another study evaluated cytochrome P450 2D6 (CYP2D6) status and HOXB13:IL17BR ratio together in a cohort consisting of ER-positive, lymph node-negative patients treated with adjuvant tamoxifen. It was reported that women with both decreased CYP2D6 metabolism and a high HOXB13:IL17BR ratio were at greatest risk and had the shortest survival [23]. Another study demonstrated that a five-gene molecular grade index (MGI) (*BUB1B*, *CENPA*, *NEK2*, *RACGAP1*, *RRM2*) + HOXB13:IL17BR+breast cancer index (BCI) were associated with risk of death among ER-positive, lymph node-negative breast cancer patients not treated with adjuvant chemotherapy [24]. These studies suggest that HOXB13 expression may be associated with breast cancer biology, particularly in the context of treatment response. Very limited studies focused on the impact of activating *HOXB13* mutations for breast cancer risk and did not find an association with *HOXB13* G84E mutation and breast cancer risk [1–3]. On the other hand, a study reported an association with *HOXB13* G84E mutation and *BRCA1/2* wild-type familial breast cancer in patients who were not of Ashkenazi Jewish ancestry [14]. In our cohort, *HOXB13* variants demonstrated a distinct distribution pattern across hormone receptor subgroups, which may suggest a potential association that warrants further investigation (Table 1). Hormone-negative patients (*n* = 3) carried the c.269del, c.766 T > C, and c.309_311del variants. Among hormone-positive patients (*n* = 8), two carried c.309_311del, one had the c.404 G > A missense variant, and the majority (five patients) carried the c.728 A > G missense variant (p.K243R), which appears to be the most frequently observed variant in this subgroup. The identification of these novel variants provides preliminary observations that may guide future studies investigating the potential clinical relevance of *HOXB13* variants in breast cancer.

Beyond variant frequency and distribution, evaluation of familial segregation patterns provided additional insights into the potential clinical relevance of these variants. Targeted segregation analysis of the identified *HOXB13* variants in available family members revealed several clinically meaningful inheritance patterns. The c.404 G > A missense variant was observed in a patient with high-grade HER2-positive invasive ductal carcinoma and in her mother, who had breast cancer at age 40, providing a suggestive example of co-occurrence in affected first-degree relatives. The recurrent c.728 A > G variant displayed multiple noteworthy familial configurations: it was detected in the father of one hormone receptor–positive breast cancer patient who was affected by prostate cancer, in line with the established relationship between *HOXB13* and prostate cancer susceptibility. In another family, the same variant was present in a maternal aunt and cousin with breast cancer, while also being found in the unaffected mother, suggesting familial clustering with incomplete penetrance; and in further cases, the variant was identified in unaffected fathers, including one family without a known cancer history, again supporting reduced penetrance. Likewise, the c.309_311del in-frame deletion was found in a patient with low-grade DCIS, her sister, and her niece who developed breast cancer at age 35, supporting a potentially relevant familial aggregation pattern. Additional c.309_311del-positive families demonstrated maternal or paternal transmission through unaffected carriers, despite reported breast cancer in more distant relatives or even in the absence of an overt family history, further reinforcing the possibility of incomplete penetrance. Finally, the c.766 T > C missense variant was identified in both a patient with high-grade DCIS and her father with prostate cancer, again providing cross-cancer clinical concordance (Table 1). Collectively, these observations do not prove causality, but they do reveal a recurrent pattern of familial aggregation, cross-cancer clustering involving prostate cancer, and unaffected carrier status consistent with incomplete penetrance. In this context, our findings strengthen the clinical suspicion that *HOXB13* may contribute to breast cancer susceptibility in at least a subset of patients. Accordingly, this work should be considered a hypothesis-generating retrospective clinical study rather than a functional demonstration of gene causality.

## Conclusion

Our findings suggest that *HOXB13* variants may be of interest in the context of breast cancer genetics, with particular attention to the c.728 A > G (p.K243R) variant in hormone-positive patients. The spectrum of identified variants observed in the Turkish population differs from that reported in European cohorts (e.g., G84E), highlighting population-specific genetic profiles. Further research is warranted to elucidate the role of *HOXB13* in breast cancer initiation and progression, and to better understand its potential biological and clinical relevance. Expanding these findings across diverse populations may contribute to future research efforts aimed at improving risk stratification and understanding disease heterogeneity ultimately improving the management of complex breast cancer subtypes. Besides, novel *HOXB13* variants, particularly c.728 A > G (p.K243R), may represent candidate variants of interest for future studies; however, their clinical relevance and potential utility as biomarkers remain to be established, especially in hormone-positive patients.

This study has several strengths, including the use of a relatively large, well-characterized cohort and a systematic evaluation of *HOXB13* variants within a clinically defined breast cancer population, with particular attention to hormone receptor status. These features provide a valuable framework for exploring the potential relevance of *HOXB13* in breast cancer genetics. However, certain limitations should be acknowledged. The identified variants are currently classified as variants of uncertain significance (VUS), and their functional and clinical impact remains to be clarified. Functional validation and tumor-based analyses (such as loss of heterozygosity) were not performed in this study. Taken together, while these findings should be interpreted with appropriate caution, they provide preliminary evidence suggesting a potential association between *HOXB13* variants and breast cancer, warranting further investigation in independent cohorts and functional studies.
